# Influence of temperature and light–dark cycle on hatching of *Eylais extendens*

**DOI:** 10.1007/s10493-018-0238-y

**Published:** 2018-03-05

**Authors:** Andrzej Zawal, Aleksandra Bańkowska, Anna Nowak

**Affiliations:** 1 Department of Invertebrate Zoology and Limnology, Institute for Research on Biodiversity, Center of Molecular Biology and Biotechnology, Faculty of Biology, University of Szczecin, Wąska 13, 71-415 Szczecin, Poland; 2 Department of Plant Taxonomy and Phytogeography, Institute for Research on Biodiversity, Center of Molecular Biology and Biotechnology, Faculty of Biology, University of Szczecin, Wąska 13, 71-415 Szczecin, Poland

**Keywords:** Water mites, Hydrachnidia, Egg diapause, Embryonic development, Impact on hatching, Evolutionary adaptation

## Abstract

Little research has been done on egg diapause and the embryonic development of water mites. The aim of this study was to check the impact of temperature and periods of light on hatching of larvae of *Eylais extendens.* Three batches of eggs which were spawned on 30 July were placed at one of three temperatures (4, 10 and 20 °C) and two periods of light (7 and 14 h per day). Egg hatching (both, percentage of hatched larvae and rate of hatching) was found to differ between 4 versus 10 °C and between 4 versus 20 °C, but not between 10 versus 20 °C. The periods of light had no influence on hatching. This synchronization of hatching, enabling the eggs to emerge from diapause in the spring, could be considered an evolutionary adaptation aimed at postponing hatching of late-spawned eggs until a time allowing for completion of the full development cycle, including the parasitic larval stage.

## Introduction

Little research has been done on the embryonic development of water mites and the impact of environmental parameters on hatching time and numbers. Most research was involved with the number of eggs spawned and the hatching time specific to particular species of water mites and the environmental determinants of these processes (Davids 1973; Mayer 1985; Daszkiewicz and Zawal 2004; Włodarczyk and Zawal 2004; Martin 2010; Dzierzgowska et al. 2011; Kłosowska et al. 2011; Cichocka et al. 2015; Bańkowska et al. 2016), whereas only two studies concerned the influence of physicochemical parameters on hatching time and numbers (Rousch et al. 1997; Martin 2010). Egg diapause has often been reported for mites in general (Walter and Proctor 1999), but has been recorded much less frequently for Trombidia (Sabori and Zhang 1996; Sabori and Kamali 1999), and only a few observations pertain to water mites (Nielsen and Davids 1975; Gerecke 2002; Martin 2010; Smith et al. 2010).

The vast majority of water mite species parasitize aquatic insects. The hosts are usually Diptera species, but other hosts include Colembola, Plecoptera, Odonata, Heteroptera, Trichoptera and Coleoptera (Smith and Olivier 1976; Boehle 1996; Baker et al. 2007, 2008; Zawal and Szlauer-Łukaszewska 2012; Zawal and Buczyński 2013: Buczyńska et al. 2015; Stryjecki et al. 2015). Water mites of the genera *Hydrovolzia*, *Piersigia*, *Hydryphantes*, *Limnochares*, *Eylais* and *Hydrachna* parasitize Heteroptera and Coleoptera (Smith and Olivier 1976). *Eylais* and *Hydrachna* are the only two genera that parasitize both Heteroptera and Coleoptera; these water mites have the longest parasitic period, which allows them to survive in the parasitic larval stage in unfavourable environmental conditions, including the winter (Nielsen and Davids 1975; Zawal 2002, 2003a). Species of the genus *Eylais* may also hibernate in the form of eggs; this applies in particular to *E. extendens* and *E. infundibulifera* (Nielsen and Davids 1975).

Additionally, it has been observed that larvae spawning in late summer failed to hatch despite completing their embryonic development (fully formed larvae were visible under the egg integument). Water mites of the genus *Eylais* hibernate mainly in the form of parasitic larvae (Zawal 2003a, b, c; Davids et al. 2006). *Eylais extendens* parasitizes Coleoptera, and both small and large larvae have been recorded on hosts from April to November, which indicates that the hosts are infected over the entire growing period. This species is considered to be bivoltine and hibernates as parasitic larvae and eggs (Nielsen and Davids 1975; Zawal 2003a). Observations of interrupted development of *E. extendens* eggs laid in late summer confirm earlier suppositions regarding hibernation of eggs (Nielsen and Davids 1975), which would allow this standing-water species to survive the winter. This raises the question: what stimulus activates later development after the hibernation period, and what environmental factors affect the number of eggs hatched? We hypothesized that temperature and/or period of light affect the percentage of hatched larvae.

## Materials and methods

Eggs were used, obtained from three female specimens of *E. extendens* caught in the first half of July. The female water mites were placed in 100-ml beakers. Eggs were laid after about 2 weeks. Egg-laying of each female lasted about 1–3 days, and each female laid 1–3 batches. About 3 weeks after the eggs were laid, fully developed larvae could be seen under the egg integuments, and some of them made small movements. In another case, where 14 batches of eggs were laid by five females in the spring or early summer, the larvae hatched when they were fully developed. Now, when eggs were laid in July, development of the larvae ceased: they stopped moving and did not hatch. The integuments remained transparent, the eggs did not become mouldy. For about 5 months the eggs were stored at about 20 °C until early January, the experiment began. The water used for breeding and experiment came from the collection site and was not exchanged during the experiment.

The batches contained several hundred eggs. Each batch was divided into six groups of about 50–90 eggs. Some of the eggs were damaged during the separation into groups. The damaged eggs turned white after a few days, which clearly distinguished them from the undamaged eggs. The eggs were placed at one of three temperatures (4, 10 or 20 °C), with two groups per temperature, and two periods of light (7 or 14 h per day), with one group per temperature. The experiment lasted 14 days, after which all eggs were placed at 20 °C with 14 h of illumination.

This experiment was conducted twice, in January and in April. In the experiment performed in January no larvae hatched. All eggs were kept under normal room conditions until April and then the experiment was repeated. This time the larvae hatched. The influence of temperature and period of light on hatching percentages was tested by Mann–Whitney U test (Statistica 12 PL).

## Results

**Fig. 1:**
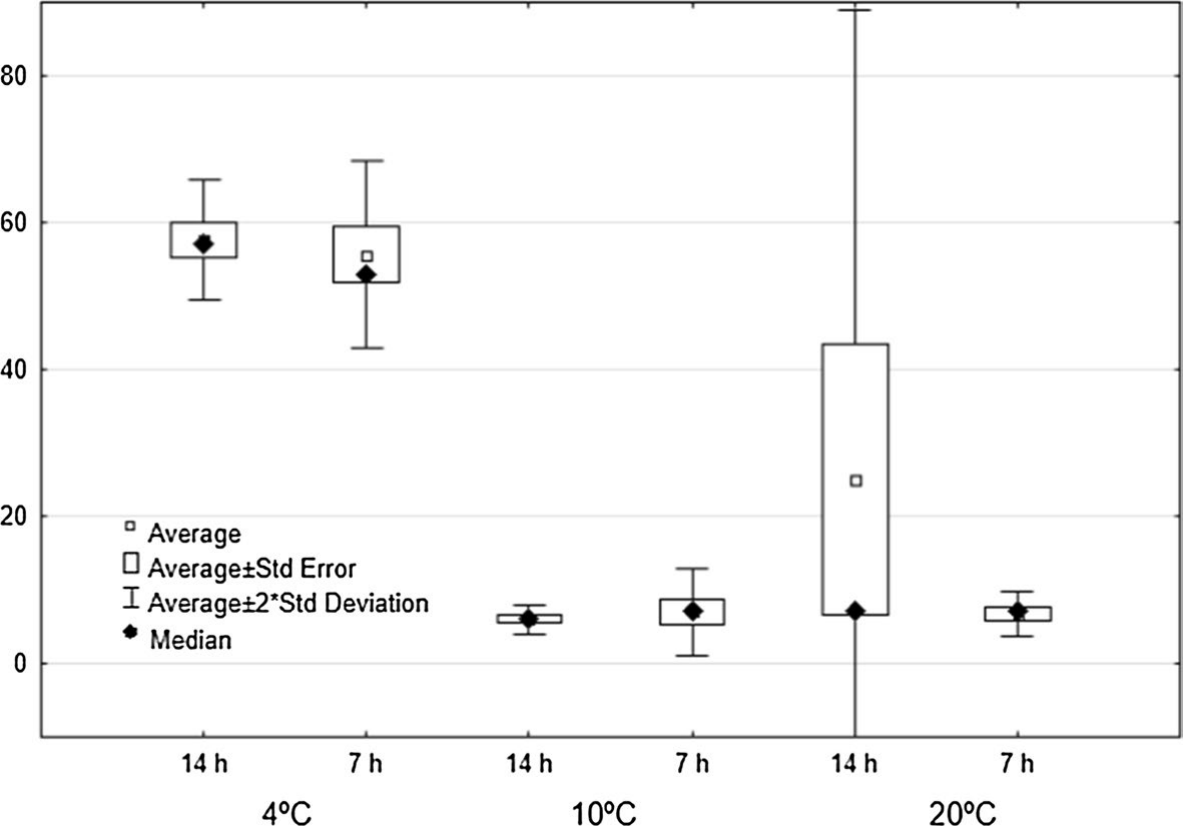
Number of hatched *Eylais extendens* eggs at three temperatures (4, 10 and 20 °C) and two light–dark cycle (L14:D10 and L7:D17)

Temperature was found to affect the percentage of hatched larvae and the hatching time. About 60% of all eggs kept at 4 °C hatched, but only 7% of those exposed to other temperatures (although in one sample kept at 20 °C ca. 60% of the larvae hatched) (Fig. 1). Pairwise comparison indicated significant differences in % egg hatching at 4 versus 10 °C (Mann–Whitney U test: Z = 2.808, *p* = 0.005) and 4 versus 20 °C (Z = 2.088, *p* = 0.037), but not between 10 and 20 °C (Z = − 0.736, *p* = 0.46). The periods of light had no influence on the hatching percentage (4 °C: Z = 0.436, *p* = 0.66; 10 °C: Z = − 0.221, *p* = 0.82; 20 °C: Z = 0.221, *p* = 0.82) (Fig. 1).

**Fig. 2:**
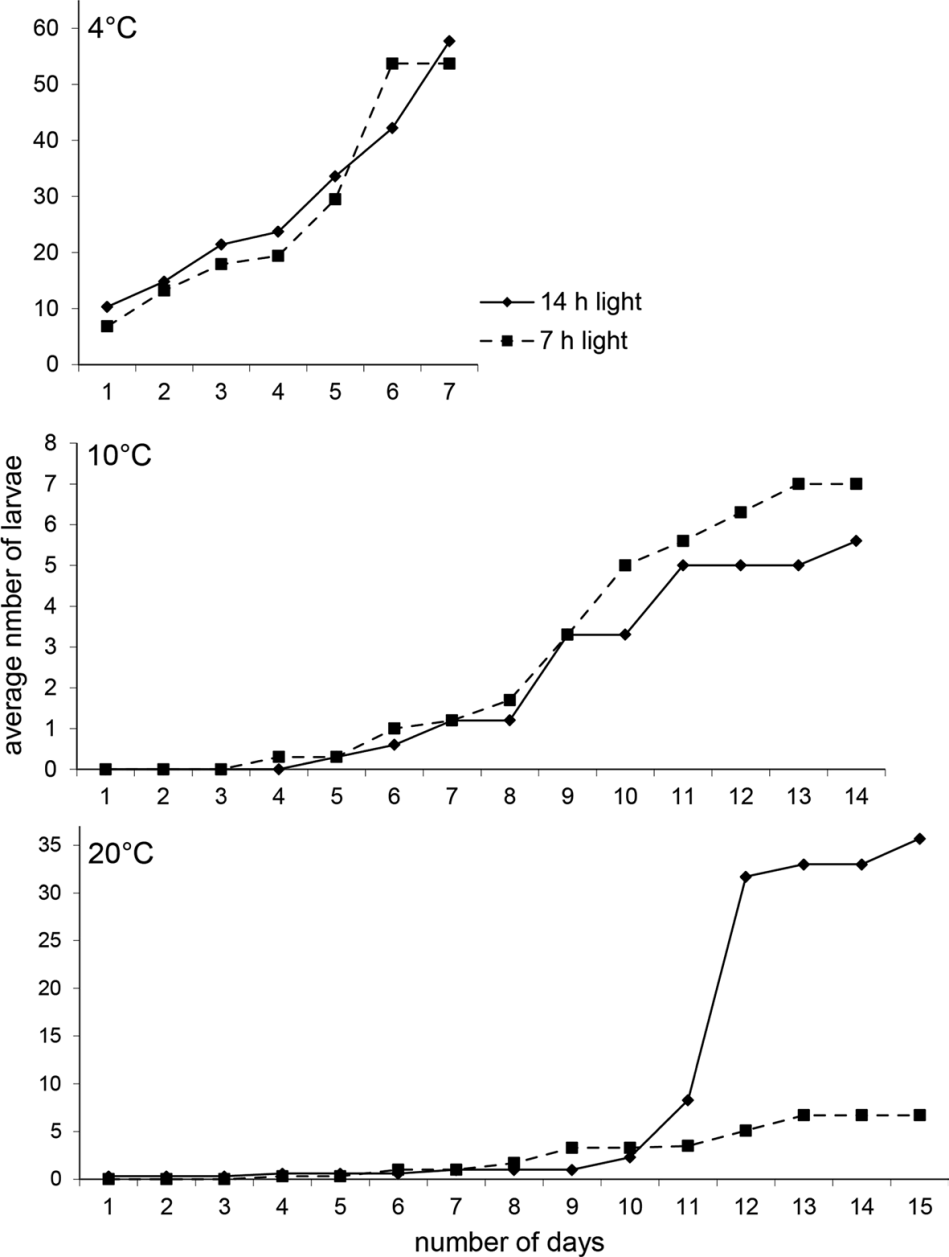
Cumulated hatching of *Eylais extendens* eggs at three temperatures (4, 10 and 20 °C) and two light–dark cycle (L14:D10 and L7:D17). Note the differences in scale on the vertical axis

The average hatching time was clearly shorter at 4 °C (about 7 days), than at 10 (ca. 14 days) or 20 °C (ca. 15 days) (Mann–Whitney U test, 4 vs. 10 °C: Z = 5.179, *p* < 0.000001; 4 vs. 20 °C: Z = 4.330, *p* = 0.000015; 10 vs. 20 °C: Z = − 0.751, *p* = 0.45) (Fig. 2). Again, the periods of light had no influence (4 °C: Z = − 0.384, *p* = 0.70; 10 °C: Z = − 0.627, *p* = 0.53; 20 °C: Z = − 0.626, *p* = 0.53).

Coming from 4 °C, a relatively large number of larvae (about 10) hatched just 1 day after the eggs had been placed at 20 °C. When hatching was complete (day 7) ca. 50 larvae had hatched. When eggs had been exposed to 10 or 20 °C, larvae began to appear after 6–8 days (Fig. 2).

## Discussion

Our results confirm the existence of egg diapause in *E. extendens*, a standing-water species—egg diapause and delay of larval hatching has previously been reported for lotic species (Gerecke 2002; Martin 2010). The results clearly indicate temperature as the factor stimulating hatching of *E. extendens* larvae. A higher percentage (about 60%) of the eggs kept at 4 °C hatched, and they hatched faster: average hatching time was about 7 days. Of the eggs which were placed at 10 or 20 °C, fewer larvae hatched (about 7%) and they hatched slower: average hatching time was about 14 days. Only the winter temperature of 4 °C increased the hatching percentage, the intermediate temperature (10 °C) had the same effect as 20 °C.

According to Martin (2010), temperature stimulates eggs to emerge from diapause in water mites inhabiting springs and streams. Eggers (1995) showed a model of egg diapause based on *Johnstoniana rapax*, in which it is controlled by a chilling period of about 90 days at 5 °C followed by a rise in temperature.

The light–dark cycle, which simulated day length, had no influence on numbers of hatching larvae or hatching time. The results were the same for both 7 and 14 h photoperiod. An effect of lighting on the hatching rate of *Sericostoma personatum* (Trichoptera) was demonstrated by Wagner (1990), who found that longer exposure to light accelerated hatching.

In our first experiment, performed in January, no larvae hatched. This indicates that, although temperature enhances the metabolic rate and is a synchronising factor, it but does not function as a stimulus inducing hatching. Also day length does not appear to be such a factor. Apparently the main factor inducing hatching is the passage of time from spawning to hatching. But we do not know what constitutes the ‘interior clock’ of water mites. Perhaps the ‘interior clock’ is the change in duration of light exposure or chilling, followed by rising temperature. This synchronization of hatching, enabling eggs to emerge from diapause in the spring, could be considered an evolutionary adaptation aimed at postponing hatching of late-spawned eggs until a time allowing for completion of the full development cycle, including the parasitic stage of the larvae (Zawal 2003a, b).

According to Lanciani (1969) and Wohltmann (2001) *Eylais* spp. are multivoltine. Böttger (1962) demonstrated a bivoltine pattern in *E. discreta* which had two types of larvae: summer ones, which start engorgement as soon as attached to their host, and autumn ones, which start engorgement after hibernating on their host. The present investigation confirms *E. extendens* as a bivoltine species, as indicated by previous literature data (Nielsen and Davids 1975; Zawal 2002), with two ways of hibernating: as a parasitizing larva or as a diapausing egg with a fully developed larva waiting to hatch.
